# Strengthening Australia’s Chemical Regulation

**DOI:** 10.3390/ijerph19116673

**Published:** 2022-05-30

**Authors:** Arlene Gabriela, Sarah Leong, Philip S. W. Ong, Derek Weinert, Joe Hlubucek, Peter W. Tait

**Affiliations:** 1 Medical School, Australian National University, Florey Building 54 Mills Road, Acton, ACT 2601, Australia; arlene.gabriela@anu.edu.au (A.G.); sarah.leong@anu.edu.au (S.L.); philipongsoowen@hotmail.com (P.S.W.O.); u6620417@anu.edu.au (D.W.); 2 Public Health Association of Australia, 20 Napier Close Deakin, Deakin, ACT 2600, Australia; j.hlubucek@bigpond.com

**Keywords:** Australian chemical policies, Australian chemical regulatory frameworks, agvet chemicals, industrial chemicals, APVMA, AICIS, chemical exposure, human health

## Abstract

Humans are exposed to a myriad of chemicals every day, some of which have been established to have deleterious effects on human health. Regulatory frameworks play a vital role in safeguarding human health through the management of chemicals and their risks. For this review, we focused on agricultural and veterinary (Agvet) chemicals and industrial chemicals, which are regulated, respectively, by the Australian Pesticides and Veterinary Medicines Authority (APVMA), and the Australian Industrial Chemicals Introduction Scheme (AICIS). The current frameworks have been considered fragmented, inefficient, and most importantly, unsafe in prioritizing human health. We evaluated these frameworks, identified gaps, and suggested improvements that would help bring chemical regulation in Australia in line with comparative regulations in the EU, US, and Canada. Several weaknesses in the Australian frameworks include the lack of a national program to monitor chemical residues, slow pace in conducting chemical reviews, inconsistent risk management across states and territories, a paucity of research efforts on human health impacts, and inadequate framework assessment systems. Recommendations for Australia include establishing a national surveillance and chemical residue monitoring system, harmonizing risk assessment and management across jurisdictions, improving chemical review efficiency, and developing regular performance review mechanisms to ensure that human health is protected.

## 1. Introduction

Every day, humans are exposed to chemicals, which are elements or compounds that are naturally occurring or manmade, through inhalation, ingestion, direct contact, and even placental transfer which affect the unborn child. In this paper, we use the term chemical to refer to compounds manufactured for use in industry, agriculture and farming, and exclude those used as human and veterinary medicines. 

Whilst chemicals are essential for everyday practices, many pose a threat to human health and the environment. Exposure to certain chemicals has been associated with a range of conditions such as asthma, chronic obstructive pulmonary disease, autoimmune disease, cancer, neurological impairment, infertility, and birth defects [1]. According to the Global Burden of Diseases, Injuries, and Risk Factors Study (GBD) 2019 [2], approximately 5.67% of total disability-adjusted life years (DALYs) in Australia in 2019 were attributable to environmental and occupational risk factors, a number which is likely to be underestimated [3]. Therefore, chemical exposure and effects monitoring and regulation remain vital to protecting the public’s health and wellbeing, especially given the continuous increase in global chemical production which can lead to further exposure and unforeseen risks. Within this global context, this paper focuses on current Australian chemical regulations.

In Australia, importation, manufacture, and use of chemicals are regulated at the national level, under four schemes, each focused on particular chemical groups and uses [4]. These include the Therapeutic Goods Administration (TGA), Food Standards Australia New Zealand (FSANZ), Australian Pesticides and Veterinary Medicines Authority (APVMA), and the Australian Industrial Chemicals Introduction Scheme (AICIS), formally known as the National Industrial Chemicals Notification and Assessment Scheme (NICNAS). The scope of this review covers the regulation of agricultural and veterinary (agvet) chemicals and industrial chemicals, introductions of which are regulated by the APVMA and AICIS, respectively, and their risk management for the protection of people and the environment. Australia’s chemical regulation has evolved over the past decades under the jurisdiction of different government departments, in a tight fiscal environment with poor coordination between the different agencies involved. Consequently, regulatory frameworks in Australia have been described as fragmented, inefficient, and most importantly, inadequate in failing to prioritize human health [5,6]. They have been criticized for not applying the precautionary principle, and misplacing the burden of proof for safety onto entities other than the manufacturer, following chemical introduction to market. Moreover, there is a paucity of data concerning the quantities of chemicals in the environment, the effects from individual and multiple different and cumulative exposures, and the potential risk of adverse health effects [5,6]. To address these shortcomings, a significant reform of Australian chemical regulatory frameworks has been underway with progress in implementation. The agvet chemicals regulatory framework is currently under review and is due for reform over the next few years [6], whilst NICNAS has been recently superseded by the AICIS with its effectiveness yet to be assessed [7]. In order to ensure that the emerging regulatory arrangements are going to best serve to reduce chemical exposure and adverse effects on human health, an evaluation of these frameworks and a comparison with international standards are necessary.

## 2. Methods

This project sought to address the following questions:

1. What are some of the risks to human health from exposure to agvet and industrial chemicals, and are there any specific vulnerable populations?
2. Are current Australian policies and regulatory frameworks sufficient to safeguard human health against chemicals?
3. What policy and regulation improvements might be needed to better safeguard Australians’ health from the introduction and use of chemicals?
4. What are the priorities for action and the consequences of inaction?

We undertook a narrative review of contemporary Australian agvet and industrial chemical regulation with respect to the protection of human health and comparative international chemical regulatory frameworks in the EU, US, and Canada. We proposed a hazard risk model which describes the interaction between chemicals in the environment, their potential for exposure to humans, and harm to human health. From this, we identified criteria to assess Australian chemical regulatory frameworks and compared them to regulations in the EU, US, and Canada. Gap analysis was performed to identify key deficiencies, from which we proposed several recommendations to strengthen existing Australian chemical regulatory frameworks.

This narrative review includes both peer-reviewed and gray literature. Peer-reviewed literature was sourced from SCOPUS, PubMed Clinical Queries, PubMed MEDLINE, PubMed TOXLINE, Web of Science Core Collections databases, and Google Scholar. Gray literature sources included websites, reports, consultation submissions, policy statements, guidelines, and other formal publications. These were obtained from a variety of stakeholders including governments and their departments, regulators, industry, and organizations, including public health organizations and communities. Inclusion criteria were all study designs and article types, in the setting of developed countries, published in the last 20 years. Exclusion criteria were articles written in a language other than English. Article titles and abstracts were searched in relevant databases or search engines using the following terms: ‘Agvet’, ‘APVMA’, ‘AICIS’, ‘CEPA’, ‘Chemical Databases’, ‘Chemical Frameworks’, ‘Chemical Monitoring’, ‘Chemical Policies’, ‘Chemical Regulations’, ‘Chemical Regulation Reviews’, ‘Chemical Regulators’, ‘Chemical Regulatory Bodies’, ‘CMP’, ‘ECHA’, ‘Environmental Health’, ‘Hazardous Chemicals’, ‘Hazard Risk Model’, ‘International Conventions on Chemicals’, ‘IChEMS’, ‘IPCheM’, ‘NICNAS’, ‘Population Health’, ‘REACH’, ‘Regulation Enforcement’, ‘Risk Assessment’, ‘Risk Reduction’, ‘TSCA’, and ‘US EPA’.

We acknowledge the limitations of the narrative review which include a lack of predefined protocols, selection bias, and subjective bias of the authors. Much of the policy aspects of the review were informed by gray literature, and there were limited peer-reviewed articles specific to some of the research questions posed. To address this, we have considered sources from a variety of stakeholders and have referred to other comparable international regulators.

No ethical issues were applicable. All sources are in the public domain or accessed through Australian National University institutional access.

The following sections of the paper respond to each of the questions posed above.

## 3. Risks to Human Health from Chemical Exposure, Particularly for Vulnerable Populations

This section provides a brief outline of the risks to health to establish the importance of robust regulatory frameworks.

### 3.1. Human Health Effects of Chemicals

#### 3.1.1. Polyfluorinated Chemical Risks

Per- and poly-fluoroalkyl chemicals (PFAS) are from a diverse group of fluorinated compounds manufactured since the 1950s [8]. Owing to their hydrophobic and oleophobic properties, PFAS have been widely used in consumer products such as disposable food packaging, cookware, outdoor gear, furniture, and carpet. The highly stable carbon-fluorine bond in PFAS prevents them from degrading quickly and their accumulation in the environment poses concerns for human health. Numerous studies have demonstrated that PFAS exposure and immune function have significant associations. The strongest metabolic disorder linked to PFAS exposure is dyslipidemia, a condition that results in an abnormal amount of lipids (e.g., triglycerides, cholesterol, and phospholipids) in the blood. Consequently, many countries have started banning its use. The association with malignancy has been demonstrated; however, this is limited to manufacturing locations with extremely high exposures. There is a lack of evidence linking PFAS exposure to neurodevelopment and associated disorders. Additionally, there are plausible links between perfluorooctanoic acid (PFOA) and several conditions such as hypercholesterolemia, thyroid disease, gestational hypertension, ulcerative colitis, kidney cancers, and testicular cancers [8,9].

#### 3.1.2. Pesticide Risks

Pesticide use is increasing due to the industrialization of the agriculture sector. Humans can be exposed to pesticides by skin contact, ingestion, or inhalation. Diuron or DCMU (3-(3,4-dichlorophenyl)-1,1- dimethylurea) is a broad-spectrum residual herbicide commonly used in Australia that works by disrupting plant photosynthesis. Between 2003 and 2006, more than 8394 tons was applied annually in Australia. Exposure to Diuron may have negative effects on human fetal development and subsequent health. Organophosphorus pesticides such as malathion, parathion, and dimethoate have been recognized for their endocrine-disrupting potential. They have also been associated with a number of cellular abnormalities such as cholinesterase dysfunction, reduction of insulin secretion, disruption of normal cellular metabolism, genotoxicity, and mitochondrial dysfunction, the latter of which generates cellular oxidative stress which affects nervous and endocrine systems [10,11,12]. Glyphosate is another broad-spectrum herbicide commonly used in agriculture; however, data concerning its effects on human health are limited and contested [13,14].

#### 3.1.3. Halogenated Compound Risks

Brominated and chlorinated flame retardants have caused much concern due to the adverse health effects of their contaminants. Such flame retardants have been linked to endocrine dysfunction, cancer, fetal and child development, neurological function, as well as reproductive and immunological toxicity [15]. Infants and children undergo rapid growth, brain, and physiological development and therefore are more vulnerable to adverse effects of chemical exposure which have compounding, downstream effects on health [16].

#### 3.1.4. Volatile Organic Compounds Risks

Volatile organic compounds (VOCs) are a diverse group of chemicals originating from industrial processes, internal combustion engines, building materials, cleaning products, and food preparation, with occupational and domestic exposures [17]. They are widespread and exposure to them is common [18]. The major health issues are cancers [19], asthma and related conditions [20], and the adverse perinatal outcomes of low birth weight and pre-term births [18]. Not only is airborne exposure important but exposure through vapor intrusion into buildings in both legacy “brown-field” sites and their surroundings due to sub-surface migration of VOCs in soil and groundwater [18].

#### 3.1.5. Heavy Metal Risks

Heavy metals such as lead represent another widely used group of chemicals. The elderly are particularly vulnerable to chemicals due to sustained exposure to lead either from occupational or environmental factors [21]. Long periods of lead exposure result in anemia, increased blood pressure in middle-aged and elderly populations, as well as reduced fertility in males. Lead exposure also has the potential to cause severe brain and kidney damage [15]. Furthermore, people working in occupations with high chemical exposure are particularly vulnerable. Lead dust has been shown to impose risk not only to the workers but to the family members [22]. Indigenous children, particularly in the mining communities, have been shown to have abnormally high levels of lead in their blood due to bare soil that contains lead contaminant [23]. LSES groups are more at risk of suffering the complications of chemical exposure due to both the increased likelihood of residing in areas with greater chemical exposure, as well as the increased susceptibility and poorer management of disease owing to health inequality [24].

### 3.2. Effects on Specific Vulnerable Populations

#### Vulnerable Populations

Chemicals have been associated with a range of illnesses and diseases which impact human health [25]. According to WHO, the global burden of disease attributed to chemical exposure is 45 million DALYs [26]. There are specific populations which need additional protection due to increased physiological vulnerability to chemical exposure and which lack capacity for advocacy and representation [27]. Most notable among this group are pregnant women and their fetuses, infants and children, the elderly, people residing in areas of low-socioeconomic status (LSES) including some Aboriginal and Torres Strait Islanders populations, and those in occupations with greater chemical exposure. It is important to consider the extent of chemical exposure and subsequent health effects in these vulnerable populations, both in the short and long term.

## 4. Review of Chemical Regulatory Frameworks

This section summarizes the regulatory framework for Australia with a focus on agricultural, veterinary, and industrial chemicals, because these are the more prevalent chemicals leading to risky exposures. We then summarize the regulatory frameworks of comparable industrialized nations to provide the basis for assessment of Australia’s regulations. We propose a model to demonstrate a set of evaluation criteria that we use to make the comparison between the Australian and the international frameworks. Finally, we map Australia against these criteria.

### 4.1. Regulation of Agricultural and Veterinary Chemicals in Australia

Agricultural and veterinary (agvet) chemicals are defined by a schedule within the Agricultural and Veterinary Chemicals Code Act 1994, termed the Agvet Code [28]. The code captures a broad range of chemicals which include those used in agricultural, forestry, and fishery industries; those used by veterinarians for livestock and domestic animals; and those used by consumers in households and public spaces to manage pests and diseases.

The Department of Agriculture, Water and the Environment applies the legislation which underpins the National Registration Scheme for agvet chemicals [4]. The scheme is designed to ensure such products are correctly labeled and packaged, effective against target species, safe when humans and off-target species are exposed, and do not pose a broader environmental risk. The scheme is administered by an independent statutory authority called the Australian Pesticides and Veterinary Medicines Authority (APVMA) [4].

Prospective products are evaluated, registered, and regulated by the APVMA up until the point of sale, after which control of use is managed by respective states and territories. The evaluation process involves assessing the efficacy and safety data of a product, with input from other agencies as appropriate. Many products also fall under the purview of other regulatory systems based on their application.

The regulatory framework for agvet chemicals is currently being reviewed to ensure the framework is up to date and fit for purpose. An initial issues paper published in March 2020 by an independent panel appointed by the Minister of Agriculture has provided a snapshot of the framework and has proposed several areas for reform [6]. Whilst the framework is supposed to provide an independent and technically proficient risk-based approach to chemical assessment, the paper proposes a number of deregulatory changes which appear to favor chemical access in a bid to increase the sector’s international competitiveness. This assessment was met with criticism from a number of stakeholders during the consultation process, such as environmental and public health groups, which asked for higher prioritization of human and environmental health. The final report of the review is in the drafting phase and is due to be submitted to the minister in the near future [29].

#### Proposed Directions of the Agvet Chemicals Regulatory Framework

In reviewing the framework, the panel has proposed a simplified hierarchy of objectives that will guide the future regulatory system. The primary stated objective is to protect the health and safety of humans, animals, plants, and the environment whilst providing safe and timely access to agvet chemicals. Three secondary objectives include the protection of trade, the promotion of primary industry, and the protection of animal welfare. Whilst the primary objective includes the protection of human health, achieving this with the view of streamlining the regulatory framework and reducing regulatory burden, particularly in the pre-market phase, is unrealistic without compromising either of these competing aims.

In an attempt to streamline the regulatory framework and recover resources that can be focused on higher risk products, the panel has proposed to increase co-regulation and self-regulation. Stakeholder feedback has generally been supportive of this concept, provided the industry continues to assume the burden of cost, in line with other regulatory systems in Australia [30]. Shifting the regulatory burden to the industry for lower-risk products aims to free up resources for the main regulator, APVMA, to focus on the regulation of higher-risk products. However, industry players are resistant to this change unless concessions are made which remove the regulatory burden elsewhere in the product lifecycle. One concession explored is the adoption of industry-led quality assurance schemes [6]. These schemes will reduce regulatory burden and expedite chemicals access, whilst ensuring consistency among jurisdictions. It is unclear. however, if these schemes will improve safety in practice, and how non-compliance will be penalized.

These are issues this paper proposed to remove, or at the minimum streamline, the efficacy assessment of prospective products. This change was met with overwhelming criticism, as efficacy forms an integral part of the risk profile of a given chemical [30]. For example, a product with low efficacy may require the application of higher doses in the environment to reach its intended biological effect, resulting in further exposure to humans, off-target species, and the environment. Thus, it is important for regulatory changes to embody the ‘as low as reasonably achievable’ principle to minimize risk to human health. Removal of this key aspect of pre-market assessment would not only undermine the credibility of the APVMA, but more importantly, severely endanger human and environmental health by permitting the usage of untested chemicals prior to any form of post-market assessment.

Registration by reference was also proposed as a means of reducing the regulatory burden [6]. This will allow prospective products to have expedited registration by referencing existing data on the same product that is already registered overseas. Conversely, products de-registered overseas would require de-registration in Australia via this method. This proposal was met with mixed feedback, as product usage and applications vary across different countries, owing to the unique characteristics of the environment [30]. Moreover, products used in other countries, which have their own standards for safety, may not be deemed safe by Australian standards, and introducing them here would compromise human health and undermine the APVMA. However, international data from comparable overseas regulators that is scientifically sound and applicable can be informative.

Off-label chemical use has been used by the agricultural industry as a means to deal with emergent biosecurity threats [6]. Nonetheless, off-label use of chemicals can be easily abused, compromising human and environmental health. The registration process allows for emergency use criteria to be defined; however, many industry players reported a lack of clarity regarding this function. The use of compounded chemicals poses another risk to human health. The proposed new definitions of agvet chemicals will allow compounded chemicals to circumvent restrictions such as registration and adverse experience reporting, thereby allowing otherwise unacceptable residues in the environment. In addition to disapproving of these changes, some stakeholders have proposed to reintroduce the re-registration and re-approval scheme from 2014 [30]. Such a scheme will ensure registered chemicals are continuing to meet safety standards well beyond their introduction.

The proposed introduction of smart labeling has been widely supported by many industry stakeholders [30]. Smart labeling enables easy access to further information that would otherwise be impractical to include on product labels. Such information could include stability and disposal information, as well as all the constituents of a product, such as surfactants and adjuvants which can be more toxic than the active ingredient itself. Moreover, smart labeling will facilitate better recordkeeping, traceability, auditing, and compliance.

Monitoring chemical usage and compliance with safety standards are important for maintaining human and environmental health. Public health and environment groups have expressed concerns that current measures to monitor chemical usage and perform environmental residue testing are inadequate [30]. The call for framework reform presents a timely opportunity to strengthen this aspect of regulation. Detailed information regarding a particular product should be real-time, accessible, and comprehensive. Moreover, information should be available in formats suitable for public access, as well as for technical purposes such as research and epidemiology. It is also critical that products are monitored throughout their entire lifecycle as health problems may only become apparent after a longer period of exposure. Compliance is another issue highlighted in the issues paper [6]. Reporting of chemical use breaches and exceedances should be mandated. Furthermore, penalties for non-compliance need to be significant so as to deter poor practices.

Control of use has been the source of much regulatory duplication, fragmentation, and inconsistency [6]. The panel proposes to harmonize control of use across the states and territories by one of three mechanisms, that is, expanding legislation that can be applied by the states and territories, using constitutional powers of the federal government to regulate agvet chemicals, or by applying an intergovernmental agreement. Many stakeholders, however, have concerns about the difficulty of this task given the varying size, needs, and privileges of the jurisdictions [30]. Some have stated this would not be possible without significant pressure from communities and politicians. The proposal that control of use should be based on geographical and environmental regions, rather than arbitrary borders, may not be realistic in practice.

### 4.2. Regulation of Industrial Chemicals in Australia

Industrial chemicals are defined by exclusion as chemicals not used for therapeutic, food, agricultural, or veterinary purposes [7]. These include a large range of chemicals used in plastics, inks, adhesives, paints, solvents, cosmetics, soaps, and other products.

On the 1st of July 2020, a new regulatory scheme for the importation and manufacture of industrial chemicals was implemented in Australia—the Australian Industrial Chemicals Introduction Scheme (AICIS), under the Industrial Chemicals Act 2019 [31]. This replaced the previous National Industrial Chemicals Notification and Assessment Scheme (NICNAS). Reforms were based on internal reviews and consultations with the public and industry.

#### 4.2.1. Changes Introduced in AICIS

##### Principle and Role of AICIS

The main principle of AICIS is Risk Proportionate Regulation, where risk is used as a basis for guiding regulatory effort at the time of introduction of the chemical [31]. NICNAS had placed more emphasis on pre-market notification and assessment. This potentially allows for a more structured, thorough approach to policies, such that relevant parties are aware of the risks motivating regulation and the potentially riskier of these are given priority. AICIS maintains the same regulatory role as NICNAS, that is, risk assessment and recommendations for risk management. However, they are also given an additional role as ‘risk manager of last resort’ [31]. This gives AICIS the power to cancel certificates or remove listings if necessary, though its primary role remains that of a risk assessor and not a manager. This addresses some stakeholder concerns about the limited role of the former NICNAS, which had little to no ability to prohibit the use of dangerous chemicals in the event that risks arise post-introduction of the chemical [5].

##### Assessment of New Industrial Chemicals

AICIS pre-introduction assessment efforts have been refocused on chemicals posing higher risks [31]. Additional emphasis has also been placed on post-introduction monitoring and evaluation. Instead of concentrating on the properties of the chemicals, their risks, including hazards and exposures, are given more weight in the introductory process. This has the potential for providing more clarity to guide efforts for the benefit of human and environmental health.

In AICIS’ categorization processes for industrial chemical introductions, exposure and hazard bands are used to determine the potential for damage, harm or adverse effects, and consequent risk to human health and the environment [32]. Criteria such as the volume, concentration, and end-use of chemicals are used to assess the likelihood of human exposure to the chemical being introduced [32]. For example, chemicals with end-use in tattoo inks or personal vaporizers will automatically be in the highest exposure band [32]. Introducers are required to provide proof that their chemicals do not have certain hazard characteristics such as carcinogenicity and developmental toxicity using test data or resources provided by AICIS [32]. Exposure and hazard bands are then combined to generate an indicative human health risk for the chemical, from very low to high risk [32]. This process also applies to risks to the environment. Chemicals of medium to high risk must be granted assessment certificates before they can be introduced [32].

There was much criticism from industry stakeholders on a large number of chemical categories (approximately 30) in NICNAS for permits, certificates, and exemptions [33]. This made the assessment process complicated and created a disproportion between the risk associated with chemicals in certain categories and the number of resources expended to assess them. AICIS’ framework limits itself to a less complex six introduction categories [31]. There is also an increased focus on industry self-regulation. Introducers are responsible for determining which level of regulation applies to their chemicals. To reduce the regulatory burden on the industry, very low-risk chemicals may be introduced without prior assessment or disclosure [31]. According to AICIS, this encourages the introduction of chemicals that are safer for human and environmental health and the threat of increased penalties is meant to deter non-compliance under the new system [31]. However, community stakeholders expressed concerns, especially in view of the lack of reporting requirements post-introduction and its implication on AICIS’ ability to conduct audits to ensure and misclassification of chemicals [34].

Additionally, AICIS has the authority to impose conditions on the introduction of higher-risk chemicals and to prohibit the introduction of certain chemicals if their risks are unable to be managed [7]. AICIS has also implemented a ban on the use of new animal test data for cosmetic ingredients.

##### Post-Introduction Monitoring and Enforcement

A prominent issue with NICNAS was its inability to track and monitor post-introduction activity and enforce compliance with regulations [33]. AICIS now has the ability to ban chemicals if absolutely necessary as a ‘risk manager of last resort’. Should future compliance issues arise, AICIS has graduated compliance powers to enable risk proportionate enforcement action [31]. In terms of tracking and monitoring, record keeping for chemical information is mandated and introducers must provide an annual declaration confirming authorization of their chemical introductions under Australian law. Similar to NICNAS, AICIS’ executive director may request information from the industry which must be fulfilled within 20 working days [31]. A post-introduction risk assessment framework has also been implemented and certain introducers now have reporting obligations for adverse chemical effects [31]. This focus on industry self-regulation and more stringent monitoring and enforcement to better safeguard human and environmental health from potentially hazardous chemicals is questioned by many public health and environment experts [34,35].

#### 4.2.2. International Chemical Regulation

##### European Union and the United Kingdoms

The EU introduced a comprehensive chemical regulation referred to as Registration, Evaluation, Authorisation and Restriction of Chemicals (REACH) in 2007, with the goal of improving human and environmental health, whilst increasing international competitiveness of the EU chemicals industry [36]. REACH places the burden on the industry to collect chemical safety information and use this to develop risk management plans which are communicated to all users [37]. This documentation is submitted in the form of a registration dossier to the European Chemicals Agency (ECHA) which determines, in consultation with Member States, whether the information provided is adequate and the chemical is safe for use [37]. REACH has two additional arms which are used to manage risk. Firstly, the EU is able to impose restrictions on the manufacturing, marketing, and use of chemicals. Secondly, authorizations are used to ensure substances of very high concern are identified and used safely whilst promoting substitution with safer chemicals [38].

The European Commission has established an initiative referred to as the Information Platform for Chemical Monitoring (IPCheM) which serves as a decentralized access point for retrieving comprehensive monitoring data of chemicals in humans and the environment [39]. IPCheM serves to assist policymakers and scientists in risk assessment research, with contributions made by a number of agencies, national bodies, and research consortia. Participants include the European Food Safety Authority, European Environmental Agency, European Chemical Agency, National Research Council Water Research Institute, and the Flemish Centre of Expertise on Environment and Health, among others [39].

The EU is a signatory to international conventions such as the Stockholm Convention on Persistent Organic Pollutants, and the Rotterdam Convention on the Prior Informed Consent Procedure for Certain Hazardous Chemicals and Pesticides in International Trade [40]. Moreover, the EU participates in other international efforts such as the Strategic Approach to International Chemicals Management, Commission on Sustainable Development, and the WHO International Programme on Chemical Safety [40,41].

The UK left the EU on the 31st of January 2020 [42]. Under the terms of the Withdrawal Agreement, REACH continued to have effect in the UK until 1st January 2021. Since then, the UK Health and Safety Executive has been established as the UK REACH competent agency taking over the function of the ECHA [43]. Several provisions in the Environment Bill 2019–2021 have given the Secretary of State powers to amend the UK regime. Whilst some protections exist to maintain the core principles of EU REACH such as the protection of human and environmental health, certain parties have expressed concern regarding the potential for regressive changes. For the purposes of this review, only EU REACH will be included [43].

##### The United States of America

In the United States, the production and distribution of chemicals are regulated by the Environmental Protection Agency (EPA) under a number of federal statutes [44]. The majority of chemicals (over 83,000) are regulated under the Toxic Substances Control Act (TSCA) including industrial chemicals [45]. Other federal laws and particular groups of chemicals they regulate include the Federal Insecticide, Fungicide and Rodenticide Act (FIFRA) which regulates pesticides, and the Federal Food, Drug and Cosmetic Act (FFDCA) which regulates foods, additives drugs, cosmetics, and devices [46].

In 2017, the EPA introduced a new process for the assessment of chemical safety [47]. Chemical safety is evaluated in a three-stage process: prioritization, risk evaluation, and risk management, each with an associated deadline [48]. Chemicals are classified into high- or low-priority categories, with high-priority chemicals undergoing immediate evaluation [48,49]. At this stage, the EPA is able to request additional data from manufacturers [48,49]. If risks are identified, EPA may take regulatory actions including limiting and banning manufacture, processing, and distribution of relevant chemicals [48,49]. During the risk management process, opportunities are available for public and stakeholder comments and consultations [50]. The EPA also performs inspections, record reviews and information requests as part of its compliance monitoring strategy to ensure adherence to laws and regulations [51].

##### Toxic Substances Control Act

The TSCA regulates commercial and industrial chemicals and enforces the prohibition of chemicals not in the TSCA Inventory [45]. On June 22, 2016, the Frank R. Lautenberg Chemical Safety for the 21^st^ Century Act was signed to amend the TSCA [52]. This addressed a number of issues under the previous TSCA. The withdrawal of chemical approvals had been a lengthy and cumbersome evaluation process for the EPA [53]. The amendment removes some of this burden from EPA to allow more timely and efficient responses for chemical management. New substantiation requirements for certain Confidential Business Information (CBI) have also been implemented to prevent manufacturers from withholding data in their chemical applications in an attempt to keep trade secrets and save on research costs [54,55]. The EPA is also required to review all new and past CBI claims for chemical identities to determine their validity [55]. Under the amended TSCA, federal–state partnership is also promoted to reduce heterogeneity in legislation among states [55]. 

In the 5 years following June 2016, a number of milestones have been achieved under the amended TSCA. These include the enforcement of substantiation of CBI claims and finalization of rules on chemical risk evaluation [47]. In 2019, EPA completed a major update of TSCA’s chemical inventory for the first time in 40 years [47]. However, the updated TSCA still received much criticism. Although the amendment gave EPA the authority to have manufacturers conduct chemical toxicity tests and relay this data, this slowed the approval of new chemicals. By early 2017, there was a backlog of around 600 chemicals waiting for approval [53]. To address this, EPA stopped requesting data and expedited approvals with very little toxicity data, creating potential health risks [53,56]. The amended TSCA’s goal to have 10 chemical risk assessments completed by June 2020 was also not met [47,53].

##### Canada

The Canadian Environmental Protection Act, 1999 (CEPA 1999) is responsible for regulating the assessment and management of chemical substances in Canada [57]. It aims to prevent pollution and protect environmental and human health [57]. Other key legislation overseeing chemical substance use include [58]:

- Pest Control Products Act—regulates products used for the control of pests;
- Food and Drugs Act—regulates foods, drugs, natural health products, cosmetics, and medical devices;
- Hazardous Products Act—determines chemical classification standards and regulates consumer products and workplace chemicals which pose risks to users.

CEPA adopts a ‘risk-based’ approach and manages chemicals through four key activities: research and monitoring, risk assessment, risk management, and compliance promotion and enforcement [59,60]. Scientific research and monitoring are used to determine the extent of exposure and the impact of chemicals on the environment and human health [57]. Substance manufacturers are required to provide specific information for risk assessment purposes [57]. If substances are deemed or suspected to be toxic, the government can take risk management measures within a specified period including limiting or prohibiting manufacture and import [57]. These measures must be published in the Canada Gazette, which is public [57]. There are opportunities for public input on regulation throughout the entire process to encourage greater compliance rates [60]. If non-compliance is identified, CEPA will investigate and utilize enforcement tools including issuing warnings, tickets, detentions, and prosecutions [57].

The Chemicals Management Plan (CMP) was implemented in 2006 to unify all existing chemical-related federal legislations into a single strategy [58]. This plan adopted a science-based approach, aiming to establish priorities and deadlines for action on high-risk chemicals, improve research and monitoring of chemical substances, collaborate with international bodies, and inform Canadians of the potential risks of chemical substances [58]. In 2006, the government had categorized all its domestic commerce chemicals (about 23,000), becoming the first country to do so [58,61]. This process identified 4300 substances requiring further attention [61]. CMP’s primary goal was to assess the risks posed by these substances [62].

With the CMP’s sunset in March 2021, a number of conclusions and recommendations have been made on the plan’s effectiveness and future directions. The CMP has made substantial progress towards its primary target, successfully addressing the majority of the priority existing chemicals and implementing risk management measures for toxic substances [63]. However, chemical risks remain a prominent issue that will require a comprehensive chemical management program [63]. Evidence is currently limited as to whether the CMP has decreased toxic substance exposure and although the program implemented several operational efficiencies, it has yet to design an approach to evaluate its effectiveness in reducing harmful health and environmental effects from substances [63]. 

### 4.3. Hazard Risk Model and Regulatory Framework Evaluation Criteria

**Figure 1:**
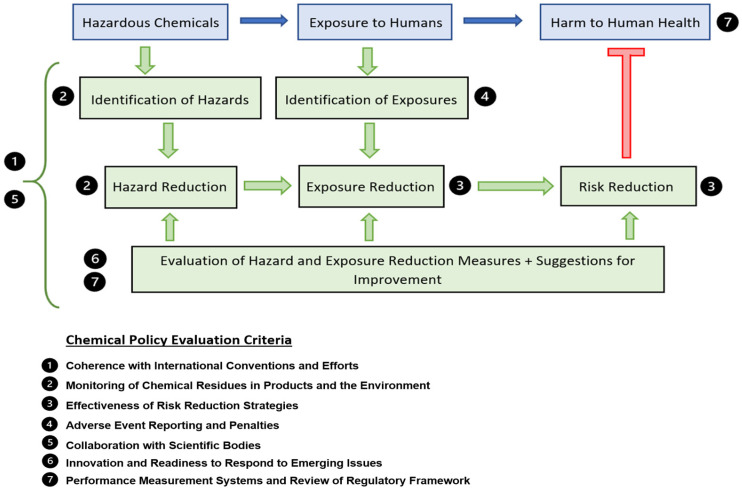
Hazard risk model describing the interaction between chemical hazard, exposure risk, and harm to human health, with corresponding chemical policy evaluation criteria.

In order to minimize the adverse effects of environmental chemical exposure on human health through policy and regulation changes, we must first examine the sequence and interaction of events leading to human harm. This can be understood qualitatively by extrapolating the health risk assessment schema used by chemical regulators and industry [64,65]. In our model (Figure 1), we describe a sequence starting with the chemical (hazard), leading to the potential for exposure to humans (risk), which may result in human harm (endpoint). This sequence can be prevented or mitigated through several strategies, namely, identification of hazardous chemicals, identification of exposure opportunities, and mechanisms to reduce both. These mechanisms themselves are also subject to evaluation and performance measurement systems to ensure they are effective in reducing risk.

We have identified several key criteria with which to compare the Australian chemical regulatory frameworks to their international counterparts. These criteria were informed by a combination of criteria used in reviews of chemical regulatory frameworks [29], as well as consideration of the components of the model and regulatory frameworks as a whole. The criteria are:

1. Coherence with International Conventions and Efforts
2. Monitoring of Chemical Residues in Products and the Environment
3. Effectiveness of Risk Reduction Strategies
4. Adverse Event Reporting and Penalties
5. Collaboration with Scientific Bodies
6. Innovation and Readiness to Respond to Emerging Issues
7. Performance Measurement Systems and Review of Regulatory Framework

The relationship between the model and the evaluation criteria is described in Figure 1. Chemical monitoring in the environment (2) is a direct function of hazard identification. Similarly, chemical adverse event reporting (4) is a direct function of exposure identification, with the effectiveness of risk reduction strategies (3) corresponding to the mechanisms used to reduce exposure. These two arms are subject to ongoing evaluation which is described by the performance measurement and review criteria (7) as well as the innovation and readiness to respond to emerging issues criteria (6). All of these interactions are strengthened through scientific collaboration and coherence with international conventions and efforts which are described by criteria (7) and (1), respectively.

### 4.4. Comparison of Regulatory Frameworks

#### 4.4.1. Coherence with International Conventions and Efforts

The APVMA participates in global joint chemical reviews under the OECD framework and collaborates with other international regulatory bodies through work-sharing arrangements which are used to inform Australian risk assessments [66]. Such bodies include the Canadian Veterinary Directorate and Agricultural Compounds and Veterinary Medicines New Zealand, among others. The recent APVMA review issues paper proposed the introduction of registration by reference, a process that allows the APVMA to expedite the registration of prospective products by referencing existing data on the same product already registered overseas [6]. This idea was met with some criticism, as chemical usage varies across different countries, owing to the unique characteristics of the environment [30]. Moreover, products used in other countries, which have their own standards for safety, may not be deemed safe under Australian standards; thus, their introduction may compromise human health.

AICIS also collaborates with the OECD Chemicals Committee and has formal bilateral cooperative arrangements with Europe, USA, and Canada, among others [67]. Information is exchanged regarding chemical assessment, emerging issues, new technologies, and regulatory models [67]. Australia is also a signatory to international conventions such as the Stockholm, Rotterdam, and Basel Conventions. However, there is a significant delay in the ratification of some conventions, particularly newer amendments of the Stockholm convention and the Minamata convention on Mercury, due to the complexity of the legal framework [68]. It is uncertain whether the AICIS reform will be sufficient to improve this [68]. AICIS also uses information on chemicals banned overseas to perform evaluations and decide if the same chemicals should be banned in Australia [69]. However, there has been public criticism on the timeliness in which this evaluation process happens [69].

The EU, through ECHA, has introduced legislation to cooperate with WHO and the UN’s international efforts, as well as international conventions to safeguard human health [40]. Some of these legislations include Persistent Organic Pollutants (POPs) regulation in conjunction with the Stockholm Convention, and Prior Informed Consent (PIC) Regulation in conjunction with the Rotterdam Convention [40]. The EU is also involved in WHO’s International Programme on Chemicals Safety (IPCS) and UN’s Globally Harmonised System of classification and labeling [40]. All of these lead to the EU becoming the global frontrunner in achieving the UN’s Sustainable Development Goal 12 [41].

Canada actively participates in and has commitments under international agreements as well as with the OECD [63]. It is also actively involved in the development of the Global Chemicals Outlook (GCO-II), a flagship publication of the United Nations Environment (UNE) on the changing global landscape of chemicals [63].

In the US, the EPA partners with multiple international organizations to ensure the protection of human health and the environment. This includes the United Nations Environment Programme under the United Nations on global environmental issues, the World Health Organization (WHO) on the protection of public health, and participation in the Arctic Council forums which promote cooperation among Arctic nations and environmental protection [70,71].

#### 4.4.2. National Monitoring of Chemical Residues in Products and Environment

In Australia, Agvet chemical residues in the environment are monitored to various extents by the government, industry, and universities. The National Residue Survey monitors chemical residues in animal and plant products; however, this effort is export-focused, and no comparable system exists for domestic products [29]. Food Standards Australia New Zealand (FSANZ) samples agvet chemical residues through the Total Diet Study; however, this effort alone is not comprehensive enough to support control of use as a whole. States and Territories are responsible for monitoring agvet chemical residues, but there is a lack of consistent processes, and only Queensland, Victoria, and Western Australia perform routine monitoring [29]. Some States rely on industry quality assurance programs that are not formally recognized or necessarily lead to compliance and enforcement. Furthermore, no national programs exist to detect pesticides in waterways, soil, and other environments [29]. Various agencies and universities perform targeted monitoring of drinking water but this again is inadequate for control of use.

Although NICNAS and AICIS chemical assessments cover both human and environmental health, the potential indirect impact of chemicals in the environment on human health is not systematically reported [69]. Australia has a number of monitoring tools such as the National Pollutant Inventory, which monitors the emission of harmful substances including industrial chemicals [69]. However, this and other monitoring tools are not systematically applied or updated [69]. There is also a lack of human biomonitoring for chemical burden among the population, which makes it difficult to evaluate the effectiveness of existing frameworks in protecting health [34].

The EU monitors chemical usage in the EU through the joint effort of EU-based agencies, as well as the national bodies of its member states [39]. Some of the types of chemicals monitored and their agency include the chemicals found in food and agriculture through the European Food Safe Agency (EFSA), chemicals released into the environment through the European Environmental Agency (EEA), and chemicals used in industrial settings through REACH [39]. This collaborative effort enables the creation of a single chemical monitoring database, Information Platform for Chemical Monitoring by the European Commission (IPCheM), which hosts all the chemical monitoring data in the EU. In addition to having a very comprehensive database, IPCheM also hosts the information on chemical monitoring with defined quality and traceability, and has a close relationship with research institutions such as the EU’s Joint Research Centre (JRC) [39].

Canada conducts a variety of environmental and human monitoring programs as well as targeted food surveys [63]. The commercial use information is also collected through reporting requirements under CEPA [63]. Notable gaps in biomonitoring include Canadians living in the Territories and First Nations peoples living on-reserve [63].

Under the Food Quality Protection Act (FQPA), EPA must ensure all pesticides used on food must meet the stringent safety standard of FQPA. Under FQPA, the EPA has to set the maximum amount of residue tolerance for each treated food. In this context, tolerance means the residue level that triggers enforcement action. The United States Department of Agriculture Pesticide Programme is responsible annually to detect residue levels that are considered to pose risk to human health. If residues exceed the tolerance level, the product can be seized by the government [72].

#### 4.4.3. Effectiveness of Risk Reduction Strategies

The APVMA dedicates significant resources to pre-market chemical assessments of all risk levels; however, post-market assessment efforts are lacking. Environmental and human health impact research is done on an ad hoc basis by research organizations and universities, rather than an ongoing effort by government and industry. Many chemical reviews have taken more than a decade to complete, and many chemicals remain in limbo after more than 15 years [29]. Furthermore, there is no clear review trigger or ongoing re-registration process for existing chemicals on the market. The current agvet chemical labeling system is also verbose and inflexible, taking away emphasis from key safety information [29]. Management of the end-of-life impacts of agvet chemicals through industry waste stewardship programs such as drumMuster and ChemClear have been considered successful [29].

Under AICIS, efforts were made to increase post-introduction monitoring. Stakeholders expressed strong concerns about the lack of reporting requirements for these chemicals and its implication on AICIS’ capacity to conduct audits to ensure misclassification of chemicals has not occurred [34]. Individual States are responsible for their own risk management, resulting in uneven risk control implementation [69]. As such, chemicals may enter the market before risk controls are in place. Although a National Industrial Chemicals Environmental Management Standard (IChEMS) is being phased into standardize risk controls across states, stakeholders are concerned that the leniency around incorporating recommended legislation into state laws may result in un-coordinated legislation [73,74]. Due to the complexity of the system and lack of monitoring data, there is limited evidence of the effectiveness of current risk reduction strategies [34]. In terms of assessment of existing chemicals, although progress is being made, over 30,000 industrial chemicals still remain untested [75].

The EU hosts a yearly evaluation of the effectiveness of its policies in regulating and restricting chemicals to safeguard human health and the environment. As per the 2020 review, the EU has evaluated that their chemical regulation through introducing REACH has halved the use of 54 hazardous chemicals subject to authorization under REACH [38]. In addition to this, the evaluation also found that there was a 97% volume reduction in total chemical usage, which demonstrates that the EU is tracking positively in terms of meeting its goal of restricting and reducing hazardous chemicals [38].

Canada’s Chemicals Management Plan (CMP) has made significant progress in assessing existing substances at pace and volume that compares favorably to international regulators [63]. Due to methodological limitations in removing confounding variables, the program does not yet have an approach to measuring and reporting on exposure and risk reduction [63]. As such, there is limited evidence for the CMP’s effectiveness in reducing the potential for exposure to harmful substances.

In the US, the TSCA authorizes the EPA to take action and address unreasonable risks to the public from chemical exposure. Under the 2016 Lautenberg amendments, the EPA must evaluate chemicals against risk-based safety standards under enforceable deadlines, with an explicit mandate to identify and assess risks to susceptible and highly exposed populations. However, chemical manufacturers are not required to provide information about chemicals under the new amendments. Furthermore, the Lautenberg amendments did not fully define what unreasonable risks are, and EPA must develop an operational definition as well as its specific risk evaluation and decision-making processes. Thus, it is difficult to determine the details of how to collect or determine the meaning of risks and how information can be obtained from manufacturers [76].

#### 4.4.4. Adverse Event Reporting and Penalties

Reporting obligations for breaches of agvet chemical use are placed on the holders of approvals, registrations, and permits. The APVMA has processed more than 20,000 reports in the last 3 years; however, most of these have been in relation to animal health concerns regarding animal chemical testing [29]. Pesticide reporting, on the other hand, has been lacking and does not reflect their ubiquitous use.

Under AICIS, certain introducers have reporting obligations for adverse chemical effects. For chemicals that have not been assessed, there is no effective reporting system as there are limited notification requirements [5]. AICIS has also made efforts to clarify secondary notification requirements which had received criticism for being unclear and cumbersome under NICNAS [5]. Regarding penalties, AICIS has only recently been given the ability to ban chemicals as well as graduated compliance powers to enable risk proportionate enforcement action [77].

EU legislation and REACH mandate that chemical use must be reported and subjected to authorization. Failure to comply can result in monetary fines and prison sentences [78]. The ECHA, as an EU agency, is classified as a community-level institution. This means that even though ECHA is governed by EU law, it does not have enforcement capabilities. Penalties for breaching legislation fall onto the EU member states to enforce; thus, there may be inconsistencies in how this is applied [78,79].

In Canada, enforcement officers must be notified of environmental emergencies for specific substances and responsible persons must take all reasonable measures for the protection of public and environmental safety. CEPA has a wide range of tools to allow both a measured and significant response to non-compliance [63].

In the US, the Office of Enforcement and Compliance Assurance (OECA) goes after pollution problems via civil enforcement and criminal enforcement. The enforcement activities of OECA include water, air, and chemical hazards. OECA works with EPA regional offices, and in partnership with state and tribal governments, and other federal agencies to enforce the nation’s environmental laws [80].

#### 4.4.5. Collaboration with Scientific Bodies

The APVMA is a member of Australia’s Regulatory Science Network (RSN) which provides a forum for scientists and technical staff to discuss scientific aspects of chemical regulation in quarterly meetings. There is also a collaboration with numerous international regulatory counterparts (described previously).

AICIS is also a member of the RSN [81]. Additionally, AICIS has partnerships with state governments and academic institutions and liaises with industry, scientists, and international regulatory bodies to assure scientific quality [82]. The Inventory Multi-tiered Assessment and Prioritisation (IMAP) program, created by NICNAS as a science and risk-based framework for assessing chemicals, now continues under AICIS [83]. However, due to the volume of substances awaiting assessment and the resourcing of agencies, there is a large number of chemicals in the inventory awaiting assessment.

The EU has a strong research collaboration effort with the EU Joint Research Centre (JRC) and EU scientific journals [84]. In addition to this, the EU’s IPCheM serves as a comprehensive chemical database that is accessible for both EU and international bodies as well as researchers for chemical research purposes [39].

Canada has a strong focus on using scientific basis to assess its chemicals. A CMP Science Committee was created to contribute expertise pertaining to scientific considerations [63]. It has run numerous meetings and produced reports on priority topics to provide a strong scientific foundation to the CMP [63].

In the US, the EPA’s Office of Research and Development Research Centre develops the knowledge, assessment, and scientific tools that comprise most of the EPA’s protective standards and guidance. It has six programs that engage with outside partners, various EPA programs and offices to identify research priorities that are important for achieving EPA’s strategic goals and objectives [85].

#### 4.4.6. Innovation and Readiness to Respond to Emerging Issues

The Australian agvet chemicals regulatory system is lagging with respect to the technological advances in agricultural and veterinary industries. The system is not equipped to review and consider changes to technologies that support chemical use such as smart labeling, augmented vision, artificial intelligence decision-making systems, and autonomous chemical applicators such as drones [29]. However, smart labeling is one application for consideration in the AVPMA review.

To support AICIS’ implementation, a number of digital systems were developed including business processes related to registration, inventory management, and evaluations [67]. An Australian customization of the International Uniform Chemical Information Database (IUCLID) software was also developed in 2019–2020 to systematically exchange international data on chemicals [67]. An internal review of NICNAS identified that it did not have sufficient scope or flexibility to respond to chemical assessments and risk management requirements of varying complexity in a timely and efficient manner [5,75]. It is uncertain if AICIS reforms are sufficient to improve this.

In the EU, ECHA regularly assesses and updates the list of chemicals to be regulated and authorized. ECHA also encourages industries and manufacturers to keep their chemical registrations up to date and accepts recommendations of chemicals to be monitored from downstream users of chemical substances [36]. In the latest strategic outlook for 2019–2023 period, the Better Regulation Programme has been introduced for more comprehensive evaluation of ECHA policies [86].

Canada’s CMP has been innovative in developing and integrating new methods and tools into its core functions. It has also begun early efforts to address emerging issues and risks. Internal review has identified opportunities for more collaboration with university-based researchers to benefit from advanced facilities available in academic research settings [63].

Funding constraints and lack of incentives in the US has resulted in gaps for the development of safety technologies, processes or products. This lack of progress can be attributed to political opposition, inadequate investment of resources for testing, enforcement, and development of safer alternatives, and the inherent complexity of chemical flows, interactions, and impacts [87].

#### 4.4.7. Performance Measurement and Review

In Australia, there are no system-wide performance measurement mechanisms in place for the regulation of agvet chemicals. Existing performance measures are operational in nature and focus on output rather than outcomes, such as the protection of human health, and others that align with the key principles of the regulatory framework [29]. There have been 24 reviews of the agvet chemicals regulatory framework since the introduction of the National Registration Scheme in 1990. However, many changes arising from these reviews have been incremental and have not addressed the fundamental concerns of the system as a whole [29]. The current review is looking thoroughly at the system from the ground up. The latest detailed APVMA review, finalized on March 2021 and passed into legislation on 10 December 2021, proposes to address many of these issues.

As AICIS was implemented recently and is still in transition, there have been no formal performance evaluations as of yet. AICIS intends to report its performance annually using criteria in accordance with the Department of Health Annual Report [88]. Under NICNAS, self-assessment reports were also published using the Regulator Performance Framework established in 2015 [68,89]. However, the lack of human biomonitoring data collected makes it difficult to truly measure performance with regard to the effect on human health [34,69]. It is uncertain to what extent Australian States and Territories have formal mechanisms in place to evaluate the effectiveness of existing risk management measures [69].

Since the introduction of REACH in 2007, there have been 13 yearly reviews conducted to evaluate the effectiveness of the policy, and how the goals are being met. Internal policy audits by the EU commission are also carried out [84]. In addition, the REACH Exposure Expert Group (REEG) works on exposure assessment for human health and the environment [90]. However, the REEG is informal and independent, with members expressing their own expert opinions which are not always unanimous. In addition, the REEG does not have any authority; thus, advice and suggestions are non-binding, and legislation passed will depend on the EU Commissioners who may not be experts in chemical regulation [90].

Canada’s CMP has a well-developed performance measurement infrastructure [63]. However, generating basic program information in a timely manner remains a significant challenge [63]. At each of the three phases of the CMP, internal evaluations were performed to examine its relevance and performance and explore future needs after its sunset [63].

In the US, the EPA determines good performance measures through three criteria, namely, meaningful, credible, and practical [91]. Despite this, the EPA’s lack of transparency makes performance review difficult [92].

Table 1 summarises the comparison between Australian and comparative international chemical regulatory frameworks across the seven evaluation criteria and highlights the gaps identified in the Australian frameworks.

## 5. Recommendations for Policy and Regulatory Improvements

The following recommendations to improve Australia’s chemical regulatory system respond to the third and fourth questions posed initially. Because agvet and industrial chemicals regulation rest with two organizations and despite the overlap, for ease of implementation planning the recommendations are made in those separate regulatory domains. 

### 5.1. Strengthening Australia’s Agvet Chemicals Regulation

- Establish a National Domestic Produce Pesticide Residue Monitoring Program Prioritising High Risk Agricultural Zones and Water Catchments as a Means of Optimising cost [29] in Line with the EU, US, and Canadian Government-Led Systems.

Such a program would be an extension of resources and outputs of the National Residue Survey and the Australia Total Diet Study and other industry residue monitoring programs such as FreshTest. Such a program would not only benefit assessments of effects on human health, but also that of ecosystem health and water safety.

- Information Collected through this Program Should then Be Readily Available in Real-Time to All Stakeholders, Including the Public, in An Appropriate form as a Means of Instilling Confidence in Australia’s Chemical Regulatory Framework.

Implementation of well-defined review triggers, such as deregistration by an international regulator, or reaching a threshold number of validated adverse event reports, as well as scheduled periodic reviews backed up by a re-registration scheme similar to the EU, US, and Canada [29]. This could build upon existing mechanisms such as codes of practice, work health and safety risk management plans, pesticide spray records, and industry waste stewardship schemes.

- Establish A National Licensing Framework to Regulate Occupational Agvet Chemical Exposure Activity.
- Create Training Standards, Especially for Restricted Chemical Products.
- Adopt smart chemical product labels which are machine-readable, to enable communication of key information such as chemical safety properties, safe use measures, first aid, and safe disposal requirements.
- Revise the current APVMA adverse events reporting system beyond animal health concerns, and integrate it better with jurisdictional reporting of other agvet chemicals such as pesticides [29]. Include residue monitoring.
- Implement a National Adverse Chemical Events Reporting System with Reports by Chemical Registrants, Users, Regulators and The General Public Being Funneled to A Single Entity and Which Are Accessible by The Public Once Validated.
- Additionally Develop a National Surveillance System to Link Together Fragmented Data Sources in The Current Regulatory Framework [29]. Such Inputs Would Include Data from Sales, National Residue Monitoring Programs, Chemical Use, Industry Quality Assurance Programs, Compliance, Adverse Events Reported, As Well As Associated Reports, Research and Decisions from International Regulators

### 5.2. Strengthening Australia’s Industrial Chemicals Regulation

- Establish biomonitoring Of Industrial Chemical Residues and Exposure with A Continuous Funding Stream Similar to The Chemical Body Burden Monitoring Programs in Europe, USA, and Canada [34].
- In Parallel, Establish a Comprehensive National Monitoring Database to Assist in Identifying Exposure Trends Over Time and by Geographical Regions and Enable Detection of Populations That May Have Increased Exposure and Risk of Adverse Effects.
- Ensure Consultation and Engagement of Stakeholders Continues with the Ongoing Monitoring of the Effectiveness of the National Data Collection and Regulatory Effort, Including Measurements to Ensure System Funding Is Adequate [88].
- Under the recently Introduced National Industrial Chemicals Environmental Management Standard (Ichems), Specify Standards for States and Territories To Incorporate Into Their Legislation To Facilitate Nationally Harmonized Risk Management and Develop a Robust System of Measurable Indicators.

IChEMS to develop performance metrics to enable the industry to show that the standards are being met [69]. 

### 5.3. Recommendations for Strengthening Both Systems where Overlap Exists

- Continue Monitoring to Determine If Recent Reforms to Agvet and Industrial Chemical Regulation Will be Sufficient to Facilitate Ratification of International Conventions in A Timely Manner.
- Systematically Report the Potential Indirect Impacts of Chemicals in The Environment on Human Health [69].
- Make clearer Distinctions Between Risks from Consumer Products and Risks to Humans Exposed to Agvet and Industrial Chemicals Through the Environment.
- Improve Resourcing to Aicis (From Government and Industry) to Enable the Inventory Multi-Tiered Assessment and Prioritisation (Imap) Program to Accelerate Assessment of the Large Number of Unassessed Chemicals Currently in Use.
- Establish a Performance Measurement Structure to Ensure That Risk Reduction Strategies are Effective in Protecting Human Health and the Environment. This Might Include Aicis and Apvma Conducting Reviews Involving Comparison with Other International Regulatory Bodies and Explore Adoption of Strategies Proven to Work Overseas and Regular External Audits by Experts on Top Of Regular Self-Assessments.
- More Timely Ratification of International Conventions.
- Establish clear Reporting Obligations with Proportional Penalties for Breaches.

## 6. Conclusions

Current chemical exposures are adversely affecting human health. This is particularly the case for vulnerable populations: the young, aged, pregnant women and their fetuses, and people in lower socioeconomic circumstances particularly Indigenous peoples. 

Both the Australian agvet and industrial chemicals regulatory frameworks require significant reform and improvement in order to bring them in line with comparative international regulators in the EU, US, and Canada. Improvements of the Australian system (detailed above) include a national chemical residue monitoring program in domestic produce and the environment, resourcing and system redesign to more rapidly review chemicals, creating national policy to ensure consistent surveillance, reporting, and risk management across jurisdictions, increased biomonitoring and focused research on human health impacts of chemical exposure, and improved performance review systems. Data from these programs and efforts should be integrated into a comprehensive national system that will inform all aspects of chemical regulation. All stakeholders, including community members, product users, public health and environmental health organizations, the regulator, governments, and industry need to actively participate in the governance and oversight of the system. 

The recent changes to AICIS and the proposed changes to the APVMA have been guided by government priorities to reduce the regulatory burden on the industry. This may increase the risk of chemical exposure to people and the environment, and this needs to be monitored.

A key principle of all regulation is the protection of human and environmental health. Failure of regulation increases exposures and hence increases the burden of preventable disease. Combined with other major environmental changes (climate disruption, topsoil loss, and biodiversity loss among others) accumulating chemical toxins in the environment reduce the adaptive capacity of all species including our own. 

Protection of animal and human populations from unnecessary and dangerous chemical exposures is, therefore, a central public health action.

The recommendations made here will help with this central role.

## Figures and Tables

**Table 1:** Comparison of Australian and comparative international chemical regulatory frameworks in the EU, US, and Canada across seven different criteria, including gaps identified in the Australian frameworks.

	Australia—Agvet Chemicals	Australia—Industrial Chemicals	EU	US	Canada	Gaps in Australian Regulatory Frameworks
**Coherence with International Conventions and Efforts**	Collaborates with a number of international regulators and bodies to perform joint reviews.	Cooperates with international regulators and conventions. Delays in ratification of some international conventions and evaluations of overseas chemical bans.	Cooperates with international conventions and efforts, leading the EU to be the global frontrunner in achieving the UN’s Sustainable Development Goals.	Partnering with multiple international organizations ensure protection to human health and environment.	Actively participates in international agreements and efforts, which includes the development of key international publications.	On par with comparable international examples, but could be more timely in ratifying some international conventions.
**Monitoring of Chemical Residues in Products and Environment**	No comprehensive national program to monitor residues in domestic produce and environment. Lack of consistent and timely monitoring across states and territories.	Monitoring tools are not systematically applied or updated. Lack of human biomonitoring for chemical burden among the population.	Comprehensive data collection by various EU agencies and EU member states’ national bodies. IPCheM database which contains comprehensive data on chemicals in the environment.	Lacking a comprehensive approach to monitor chemicals in products and the environment.	Conducts a variety of environmental and human monitoring programs. There are gaps in biomonitoring of a few specific populations.	Behind the EU with respect to having a comprehensive national program to monitor all chemicals which is easily accessible, adopted by all jurisdictions, and performed in regular timely intervals.
**Effectiveness of Risk Reduction Strategies**	Limited resources allocated to post-market assessment of chemicals. Human health impact research is seldom performed by government and industry.	Increased introducer self-regulation and lack of reporting requirements. Uneven implementation of risk controls. Limited evidence on effectiveness of strategies.	Effective reduction of number and volume of hazardous chemicals used.	Limited evidence in its effectiveness for risk reduction.	Assessed existing chemicals at a pace and volume that compares favourably to international regulators. Limited evidence on effectiveness in reducing exposure to chemicals.	Behind the EU in regards to timely post-market assessment and review of chemicals. Human health impact research is done at the discretion of research organisations and academia.
**Adverse Event Reporting and Penalties**	Limited reporting of inappropriate pesticide use and harms.	No effective reporting system for unassessed chemicals. Recently given basic ability to ban chemicals as well as graduated compliance powers.	Strong penalties exist however enforcement is carried out by individual EU members and may not be uniform.	OECA works closely with EPA’s regional offices and partners with state and tribal governments and other federal agencies to ensure enforcement of the nation’s environmental law.	Mandatory notification for environmental emergencies. Has a wide range of tools to allow both measured and significant response to non-compliance.	Behind the EU and Canada in terms of having an effective adverse event reporting system which applies to all chemical usage and has clear reporting obligations with proportional penalties.
**Collaboration with Scientific Bodies**	Part of the Regulatory Science Network and collaborates with international regulatory counterparts.	Part of the Regulatory Science Network and has international and academic partnerships.	Multiple collaborations with scientific bodies in the EU.	Research and Development Research Centre underpins vast majority of EPA protective standards and guidance.	Strong focus on scientific basis. CMP Science Committee contributes expertise on scientific considerations.	On par with international examples, however, opportunities for collaboration and consultation could be more frequent.
**Innovation and Readiness to Respond to Emerging Issues**	Unequipped to review newer chemical technologies used by industry.	Lack of scope or flexibility to respond to complex issues in a timely and efficient manner.	Open suggestion policy available during yearly reviews to allow for adaptation to emerging issues.	Lagging in its responsiveness to emerging issues.	Innovative in the development of tools and early response to emerging issues.	On par with international examples, but does not have the scope to respond to emerging technologies involving chemical use.
**Performance Measurement and Review**	No system-wide performance measures which focus on outcomes such as human health. No fundamental review opportunities until recently.	Annual Reports under NICNAS with intention to continue under AICIS. Uncertain if states and territories have formal evaluation mechanisms in place.	Yearly policy reviews and other internal audits by the EU commission. REEG provides specific advice and recommendations for REACH.	Difficult to assess due to lack of disclosure by the EPA.	Well-developed performance measurement infrastructure. Issues generating basic program information in a timely manner.	Behind the EU with regards to lacking yearly opportunities to review the regulatory systems at a fundamental level.

## Data Availability

Not applicable.
